# Synthesis and antibacterial activities of Ag-TiO_2_/ZIF-8

**DOI:** 10.3389/fbioe.2023.1221458

**Published:** 2023-07-27

**Authors:** Siqi Bao, Shuanghui Sun, Lin Li, Lei Xu

**Affiliations:** School of Chemistry and Environmental Engineering, Changchun University of Science and Technology, Changchun, China

**Keywords:** antibacterial activity, bioactive organic materials, *Escherichia coli*, *Bacillus subtilis*, Ag-TiO_2_, ZIF-8

## Abstract

In recent years, massive bacterial infections have led to human illness and death, reminding us of the urgent need to develop effective and long-lasting antimicrobial materials. In this paper, Ag-TiO_2_/ZIF-8 with good environmental friendliness and biological antibacterial activity was prepared by solvothermal method. The structure and morphology of the synthesized materials were characterized by XRD, FT-IR, SEM-EDS, TEM, XPS, and BET. To investigate the antibacterial activity of the synthesized samples, *Escherichia coli* and *Bacillus subtilis* were used as target bacteria for experimental studies of zone of inhibition, bacterial growth curves, minimum bactericidal concentration and antibacterial durability. The results demonstrated that 20 wt.%Ag-TiO_2_/ZIF-8 had the best bacteriostatic effect on *E. coli* and *B. subtilis* under dark and UV conditions compared to TiO_2_ and ZIF-8. Under the same conditions, the diameter of the inhibition circle of 20 wt% Ag-TiO_2_/ZIF-8 is 8.5–11.5 mm larger than that of its constituent material 4 wt% Ag-TiO_2_, with more obvious antibacterial effect and better antibacterial performance. It is also proposed that the excellent antibacterial activity of Ag-TiO_2_/ZIF-8 is due to the synergistic effect of Ag-TiO_2_ and ZIF-8 under UV light. In addition, the prepared material has good stability and durability with effective antimicrobial activity for more than 5 months.

## 1 Introduction

In recent years, the proliferation of microorganisms such as bacteria and viruses in the environment has posed a serious threat to the ecosystem and human health. Antibacterial materials are functional materials that can kill harmful bacteria or inhibit the growth and reproduction of harmful bacteria. The antibacterial effect of antimicrobial materials is generally evaluated by factors such as the diameter of the inhibition circle, the concentration of the minimum inhibition circle, and the bacterial growth curve. *E. coli* and *Bacillus subtilis* are two typical bacteria that are widespread in daily life and can cause health hazards such as abdominal pain, diarrhea, etc. However, the antimicrobial agents currently in use have disadvantages such as short expiration dates, high consumption, and specific hazards to the surrounding area, either by themselves or as by-products, which greatly limit the practical application. With the increasing human requirements for environmental health and recognition, the research and development of long-lasting, stable and environmentally friendly antimicrobial agents has become one of the hot spots of concern for many scholars.

TiO_2_ has become a promising photocatalytic antibacterial agent due to its excellent characteristics such as green, stable, broad-spectrum antibacterial and simple preparation (Hayashi et al., 2020; Rodriguez-Gonzalez et al., 2020). However, it also has many drawbacks, such as: weak light capture ability and small specific surface area, which limit its application in the field of photocatalytic antibacterial. To compensate for these drawbacks and obtain highly active photocatalytic antimicrobial agents, metal loading (e.g., Ag, Pt and Au) was used to modify the TiO_2_ (Abadikhah et al., 2019; Ponomarev et al., 2019; Nasim et al., 2020; Xue et al., 2020). As we all know that silver nanoparticles are widely studied and used thanks to their excellent and long-lasting antibacterial activity as well as low induced drug resistance (Xu Y. et al., 2020). Dong et al. reported Ag-TiO_2_ nanocomposites prepared by dielectric barrier discharge (DBD) cold plasma treatment could effectively inhibit the growth of *E. coli* and *Staphylococcus aureus* (Dong et al., 2019). The surface modified by Ag/TiO_2_ nanoparticles prepared by Lu et al. was able to remove *E. coli* and *B. subtilis* under visible light induction (Lu et al., 2019). However, Ag/TiO_2_ still has disadvantages such as aggregation phenomena and limited antibacterial activity.

Metal organic frameworks (MOFs) are porous crystalline materials with a periodic network structure composed of metal centers (metal ions or metal clusters) and bridging organic ligands, which have attracted increasing attention due to their large specific surface area, ease of separation, and structural and functional diversity (Zhu et al., 2019; Chen et al., 2021). Among the MOFs, ZIF-8 is a member of the zeolitic imidazolium framework (ZIF) family, which has been widely used in adsorption, catalysis, energy storage and separation processes due to its high specific surface area, special pore structure and good thermal stability (Zheng et al., 2020; Ahmad et al., 2021). Because of the central metal ion Zn^2+^ with antibacterial activity, ZIF-8 can also be used as an antibacterial agent (Xu W. et al., 2020). Combining MOFs with inorganic substances can effectively improve the antibacterial activity of the complexes. In this experiment, the bacterial inhibitory ability was enhanced by compounding ZIF-8 with TiO_2_ and Ag. Nabi-pour et al. tested the antimicrobial activity of ciprofloxacin/ZIF-8 against *E. coli* and *S. aureus*, and the results exhibited that the synthetic agent could well inhibit the growth of a range of microorganisms such as bacteria (Nabipour et al., 2017). Guo et al. successfully fabricated core-shell Ag@ZIF-8 nanowires, which showed significant antibacterial activity against *E. coli* and *B. subtilis* (Guo et al., 2018). Malik et al. synthesized multifunctional CdSNPs@ZIF-8 with antimicrobial activity against *E. coli* and *S. aureus* (Malik et al., 2018).

Hence, based on the above discussion, Ag-TiO_2_/ZIF-8 ternary composites with excellent biological antibacterial activity were successfully prepared by the solvothermal method. ZIF-8 was introduced into the material to increase the specific surface area, thereby increasing the contact area between the biocide and the bacteria. In addition, Ag^+^ and Zn^2+^ can promote the separation of TiO_2_ electron-hole pairs, enhance photocatalysis and generate more active substances to attack organic macromolecules in bacteria for their oxidative degradation (Younis et al., 2020; Ma et al., 2021). Then, the structure, morphology and elemental analysis of the prepared materials were studied in detail by XRD, FT-IR, XPS, TEM and EDS. Finally, *E. coli* and *B. subtilis* were selected for antibacterial activity evaluation experiments, such as inhibition zone, bacterial growth curve, minimum bactericidal concentration and antibacterial durability, and the antibacterial mechanism of Ag-TiO_2_/ZIF-8 was further explored.

## 2 Materials and methods

### 2.1 Materials

Tetrabutyl titanate [Ti(OBu)_4_], silver nitrate (99.8%, AgNO_3_), absolute ethanol (99%), glacial acetic acid (98%), zinc nitrate hexahydrate, 2-methylimidazole, N,N-dimethylformamide (DMF), dichloromethane (DCM) and chloroform were purchased from Tianjin Guangfu Fine Chemical Research Institute Co., Ltd. China. *Escherichia coli (E. coli)* and *B. subtilis (B. subtilis)* were obtained from the private collection of the Biology Department, School of Life Science and Technology, Changchun University of Science and Technology. All biological reagents were purchased from Ob-xing Biological Co., Ltd. China. Deionized water was used throughout the experiments except for antibacterial activity evaluation experiments when sterile water was used. All chemicals were used directly without further purification.

### 2.2 Synthesis of Ag-TiO_2_

Ag-TiO_2_ was synthesized by the sol-gel method using Ti(OBu)_4_ and AgNO_3_ as the titanium source and dopant, respectively. Solution 1 was prepared by adding a few drops of Ti(OBu)_4_ (8.5 mL) to absolute alcohol (20.0 mL). A certain amount of AgNO_3_, acetic acid (2 mL), pure ethanol (6 mL) and a small amount of deionized water were mixed to obtain Solution 2. After the two solutions became abrasive, solution 1 was added dropwise to solution 2 under stirring conditions until a clear sol was produced and aged for 12 h to form a gel. Then, the gel was heated to 200°C at 2°C/min, maintained 120 min and cooled under ambient condition. The final product was obtained by washing three times in deionized water, baking at 70°Ctemperature overnight and then crushing into a purple-black powder. The theoretical content of Ag among the prepared Ag-TiO_2_ was 1wt%∼6wt%. For the sake of comparison, the above-mentioned steps were repeated in the absence of AgNO_3_ to prepare TiO_2_.

### 2.3 Synthesis of Ag-TiO_2_/ZIF-8

The Ag-TiO_2_/ZIF-8 ternary composites were synthesized by the solvothermal method. ZnNO_3_•6H_2_O (0.478 g), 2-methylimidazole (0.120 g) and Ag-TiO_2_ (0.008 g) were dissolved in DMF (36 mL) and stirred thoroughly. A certain mass of 4 wt% Ag-TiO_2_ was added into the above solution and stirred well to prepare a synthetic mixed solution. Then the mixture solution was heated to 140°C at 5°C/min and held for 24 h, then cooled to ambient temperature at 0.4°C/min. The final product was washed sequentially with chloroform, DMF and DCM, dried at 50°C, and ground to a brownish-yellow powder to finally obtain 4 wt% Ag-TiO_2_/ZIF-8. In the synthesized Ag-TiO_2_/ZIF-8, the theoretical content of Ag-TiO_2_ ranged from 5 wt% to 70 wt%, and the specific modification amounts were shown in Supporting Material Supplementary Table S1. For the sake of contrast, the same procedure was followed for the preparation of ZIF-8 in the absence of Ag-TiO_2_.

### 2.4 Characterization

The crystal structure of the material was studied on a Ricoh Y-2000 X-ray diffractometer with data recorded by powder X-ray diffractometer (XRD) in the range of 10° to 90°. The composition and chemical bonding of the materials were tested by Fourier transform infrared spectra (FT-IR, IFS 66V/S, Germany). The morphologies of the materials were carried out with transmission electron microscopy (TEM, Hitachi-H600). The elemental analysis was analyzed by energy dispersive spectroscopy (EDS) attached to SEM. X-ray photoelectron spectroscopy (XPS) results of materials were determined through Rigaku 2500/PC spectrometer. (In this experiment, Mg was chosen as the target source for the XPS test.) The surface area of each material was examined from the nitrogen adsorption-desorption isotherm using the Micromeritics ASAP 2020 system.

### 2.5 Study of antibacterial activity

The antibacterial performance of the material was evaluated by measuring the zone of inhibition (ZOI), bacterial growth curve, minimum bactericidal concentration (MBC), and observing the antibacterial durability of the material. *E. coli* and *B. subtilis* were selected as test organisms and tested under dark and UV conditions. The specific experimental operations were as follows: (a) Zone of inhibition: The bacterial enrichment solution (80 μL) and the prepared material (100 mg) were put into the medium, then sealed and inverted in a shaker in a constant temperature incubator for 1 day, and the diameter of the zone of inhibition was measured with a ruler. (b) Bacterial growth curves: During the bacterial growth curve experiment, the prepared material was dissolved in fresh LB liquid medium (150 mL) and sterilized at 121°C for 20 min. After cooling, bacterial enrichment cultures (10 mL) were added and incubated at 37°C. The optical density (OD) of the bacteria was then measured using a UV-Vis spectrophotometer and the growth curve of the bacteria was plotted. (c) The MBC was obtained by preparing solid media with different amounts of preparation materials, adding bacterial enrichment, and measuring the number and condition of bacterial growth. (d) The durability of the antibacterial activity of the prepared materials was determined by observing changes in the zone of inhibition on the solid culture medium after 5 months.

## 3 Results and discussion

### 3.1 Characterization of the materials

**FIGURE 1:**
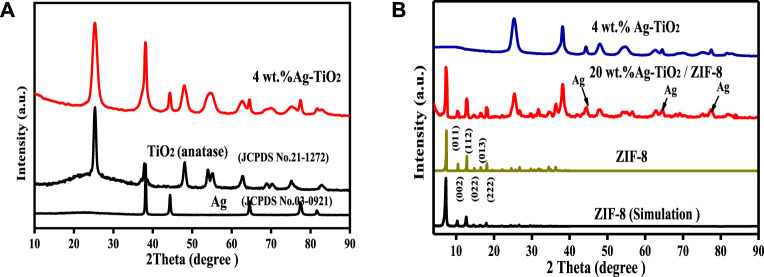
XRD patterns of as-synthesized TiO_2_, 4 wt% Ag-TiO_2_ **(A)** and ZIF-8, 4 wt% Ag-TiO_2_, 20wt.%Ag-TiO_2_/ZIF-8 **(B)**.

The crystal structure and phase constitution of as-synthesized materials were identified by XRD, and the patterns are illustrated in Figure 1.

As shown in Figure 1A, the diffraction peaks (2θ) of Ag-TiO_2_ at 25.3^o^ (101), 37.8^o^ (112), 48.0^o^ (200), 62.7^o^ (204), 68.8^o^ (116), 75.3^o^ (215) and 82.5^o^ (303) are consistent with the standard XRD data of anatase phase TiO_2_ (JCPDS No. 21-1272), showing that the TiO_2_ used as a carrier has an obvious anatase structure, and the phase structure of TiO_2_ did not change significantly after the silver modification. The other peaks of Ag-TiO_2_ at 38.2^o^ (111), 44.5^o^ (200), 64.5^o^ (220) and 77.5^o^ (311) correspond to the cubic crystal form of pure silver (JCPDS No. 03-0921). The change from baryons to singletons at 2θ ≈ 54.4° may be due to the presence Ag^0^ and AgO_x_. Therefore, the peaks of Ag-TiO_2_ matched well with Ag and TiO_2_, proving that Ag-TiO_2_ material was successfully prepared. Figure 1B suggests that the diffraction peaks of ZIF-8 is in good agreement with the previously published results (Jia et al., 2020), and the sharp diffraction peaks illustrated good crystal shape, confirming the formation of pure ZIF-8 phase. All the diffraction peaks of Ag-TiO_2_/ZIF-8 can be associated with crystalline ZIF-8 and Ag-TiO_2_, and no peaks of impurities are detected, which indicates the successful synthesis of Ag-TiO_2_/ZIF-8. Moreover, Ag-TiO_2_ doping into ZIF-8 does not affect the integrity of its crystal structure and framework.

**FIGURE 2:**
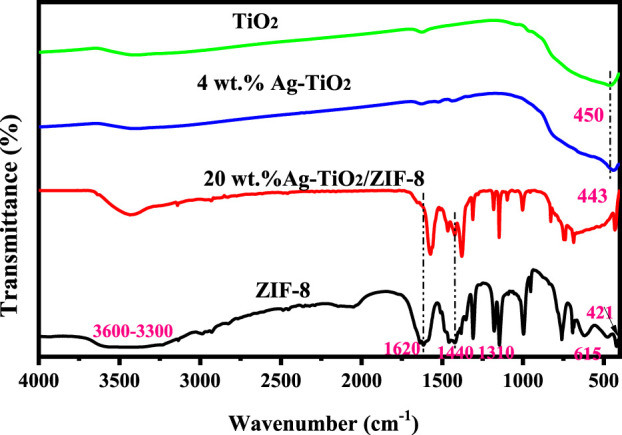
FT-IR spectra of ZIF-8, TiO_2_, 4 wt.%Ag-TiO_2_ and 20 wt.%Ag-TiO_2_/ZIF-8.

The FT-IR spectra of the prepared materials are displayed in Figure 2. The FT-IR spectra of 20 wt% Ag-TiO_2_ are shown in Supplementary Figure S1. The characteristic band of TiO_2_ at 450 cm^-1^ is related to the vibrational mode of Ti-O-Ti bond, which shifts slightly to a lower frequency (443 cm^-1^) after the silver modification (Li et al., 2019). In the spectrum of ZIF-8, the stretching pattern of the NH groups was discovered by analyzing the absorption band from 3300 to 3000 cm^-1^ (Troyano et al., 2019). The bands at 1620, 1440 and 421 cm^-1^ are attributed to the stretching modes of the C = N, C-N and Zn-N bonds, respectively (Abdi, 2020; Lia et al., 2020). The adsorption bands in the 600-1300 cm^-1^ region are attributed to stretching and bending vibrations of the imidazole ring (Li Y. Y. et al., 2020). These assignments are in common with the previously reported results (Vaidya et al., 2019). All characteristic bands of 4wt.%Ag-TiO_2_ and ZIF-8 can be observed in the spectra of 20wt.%Ag-TiO_2_/ZIF-8, indicating the successful construction of the complex. The smaller displacements at 1620 cm^-1^ and 1440 cm^-1^ may be due to the interaction of various functional groups among Ag-TiO_2_/ZIF-8.

The TEM study the morphology and microstructure of the prepared materials. The results shows that pure ZIF-8 crystal has a dodecahedral sodalite structure with many hexagonal and quadrilateral planes (Supplementary Figure S2A), which is consistent with the previous report (Bin et al., 2020). In Supplementary Figure S2B, silver granules are well scattered on the surface of TiO_2_, and this is because the radius of Ag^+^ (12.6 nm) is larger than that of Ti^4+^ (7.45 nm), causing silver ions hardly enter the lattice of TiO_2_ (Prabhu and Valan, 2020). Ag-TiO_2_/ZIF-8 consists of Ag, TiO_2_ and ZIF-8, and spherical Ag-TiO_2_ particles are observed on the surface of the ternary complex (Supplementary Figure S2C), demonstrating the modification of Ag-TiO_2_ makes no difference for the crystal structure and the integrity of framework. Furthermore, a typical EDS spectrum in Supplementary Figure S2D verifies the existence of C, O, N, Ag, Ti, Zn elements, and the silver content is 0.72 wt%. The Mapping of Ag-TiO_2_/ZIF-8 is shown in Supplementary Figure S2E–J, and it can be found that the prepared materials have good dispersion properties.

**FIGURE 3:**
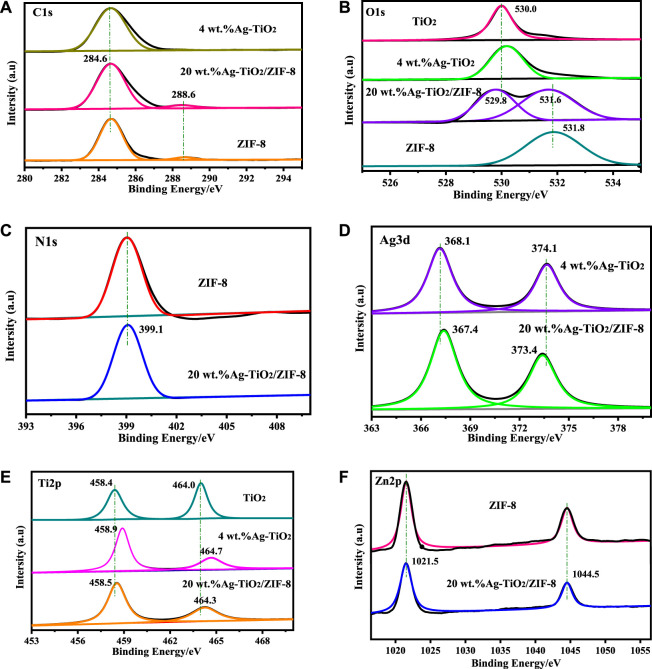
XPS: **(A)** C 1s XPS pattern, **(B)** O 1s XPS pattern, **(C)** N 1s XPS pattern, **(D)** Ti 2p XPS pattern, **(E)** Ag 3d XPS pattern, **(F)** Zn 2p XPS pattern.

The surface electronic elemental composition of the prepared materials is conducted by XPS. Supplementary Figure S3 confirms the existence C, N, O, Zn in ZIF-8, C, O, Ag, Ti in Ag-TiO_2_ and C, N, O, Ag, Ti, Zn in Ag-TiO_2_/ZIF-8. The element C is derived from the ligand of ZIF-8 and CO_2_ adsorbed on the surface of the materials in the C 1s spectrum (Figure 3A). The main peaks located at 284.6 eV and 288.6 eV of 20 wt.%Ag-TiO_2_/ZIF-8 are ascribed to C-C and C-N, respectively (Shwetharani et al., 2019; Ji et al., 2020). In comparison with the cohesive energy of O in TiO_2_ and ZIF-8, two peaks are found and the binding energy display negative shifts after loading (Figure 3B), suggesting interactions exist ternary complex. The peak at 531.6 eV and 529.8 eV correspond to O 1s binding energy of C=O and lattice oxygen of anatase phase TiO_2_, respectively (Singh and Mehata, 2019). The N 1s peak at 399.1 eV can originate from sp^2^-hybridized nitrogen (C=N-C) (Figure 3C) (Wang et al., 2020). The 6.0 eV variance between the binding energy of Ag3d_5/2_ and Ag 3d_3/2_ peak among Ag-TiO_2_/ZIF-8 (Figure 3D) is characteristic of metallic Ag 3d state, implying that Ag exists as Ag^0^ and AgO_x_ rather than Ag^+^, which is consistent with the results of XRD. In Figure 3E, the binding energy located at 458.5 eV and 464.3 eV were put down to Ti 2p_3/2_ and Ti 2p_1/2_, respectively, which is consistent with the Ti 2p spectrum of TiO_2_ (Rempel et al., 2020). By comparison, Ag-TiO_2_/ZIF-8 (Figure 3D, E) displayed small shifts, revealing Ti-O-Ag chemical bonding exists. The Zn 2p peak splits into two peaks of Zn 2p_3/2_ (1021.5 eV) and Zn 2p_1/2_ (1044.5 eV) (Figure 3F) (Ren et al., 2019), demonstrating the state of Zn element remains unaltered after synthesizing Ag-TiO_2_/ZIF-8.

Supplementary Figure S4A displays the N_2_ adsorption-desorption isotherms for 4 wt.%Ag-TiO_2_, ZIF-8 and different loadings of Ag-TiO_2_/ZIF-8. The Ag-TiO_2_ shows type IV isotherms with hysteresis loops, demonstrating the prepared material is mesoporous. The ZIF-8 belongs to type I isotherm, indicating the presence of microporous, which is in line with the results published previously (Li C. E. et al., 2020). Meanwhile, the pore-size distributions also show that ZIF-8 was mainly microporous (Supplementary Figure S4B). Between different loadings of Ag-TiO_2_ (20 wt%, 50 wt%), it was found that the increase of N_2_ adsorption slowed down with increasing loading in the region of P/P_0_ < 0.01, and a hysteresis loop appeared in the range of P/P_0_ = 0.4–0.8, which indicated that the microporous structure characteristics became less pronounced and the mesoporous structure gradually became obvious. Therefore, a conclusion could be drawn from the above discussion that Ag-TiO_2_ was attached to the surface of ZIF-8 and covered the microporous of ZIF-8. This assumption was consistent with the TEM image. Moreover, the average pore size of 20wt% Ag-TiO_2_/ZIF-8 (4.46 nm) was larger than that of ZIF-8 (3.84 nm), and the BET surface areas was smaller than that of ZIF-8 (637.9 m^2^▪g^–1^) (Supplementary Table S2), which demonstrated Ag-TiO_2_ exists on the surface of ZIF-8, confirming the conclusion of N_2_ adsorption-desorption isotherms.

### 3.2 Antibacterial activity evaluation

#### 3.2.1 Zone of inhibition

**FIGURE 4:**
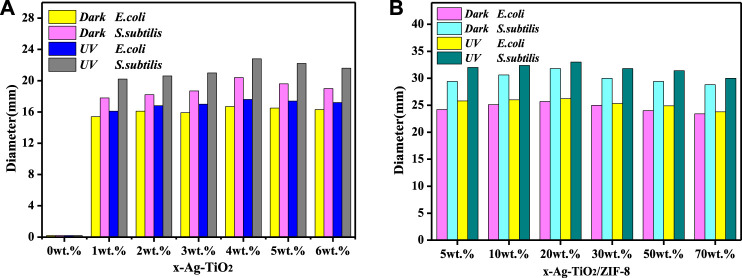
Diameters of ZOI of x-Ag-TiO_2_ **(A)** and x-Ag-TiO_2_/ZIF-8 **(B)**.

**FIGURE 5:**
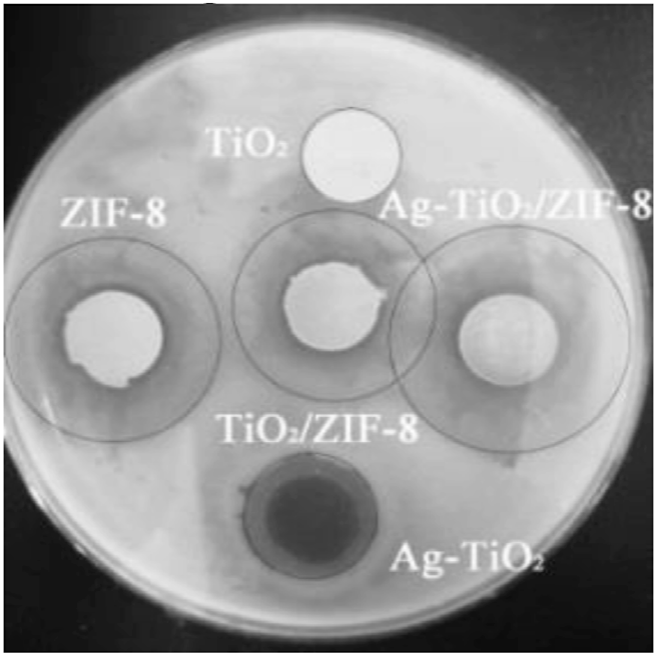
Images of ZOI of the prepared materials.

The antibacterial activity of the prepared materials was evaluated through the ZOI assay (Zheng et al., 2023), and the results are summarized as follows. Combined with Figure 4A, Supplementary Figure S5, S6 and Supplementary Table S3, the control reactions show that no antibacterial activity was observed for pure TiO_2_, while the antibacterial activity was significantly increased by the silver modification under dark and UV conditions. Moreover, the results show that the obtained 4 wt% Ag-TiO_2_ at 4% silver deposition (mass ratio to pure TiO_2_) had the best antibacterial activity against *E. coli* and *Bacillus subtilis* with ZOI diameters of 17.6 and 22.8 mm under UV light, respectively. As the degree of silver modification continues to increase, it readily agglomerates into large size silver particles, reducing its dispersibility and performance, thus weakening the antimicrobial activity of the material. Therefore, 4 wt.%Ag-TiO_2_ was chosen for further experiments. Loading studies on different amounts of 4 wt.%Ag-TiO_2_ reveals that the obtained 20wt% Ag-TiO_2_/ZIF-8 has outstanding antibacterial activity against *E. coli* and *B. subtilis* when Ag-TiO_2_ was deposited at 20% (mass ratio to pure ZIF-8), and the diameters of ZOI are 26.2 and 33.0 mm under UV light, respectively (Figure 4B, Supplementary Figure S7, S8 and Supplementary Table S4). Under equivalent conditions, the diameter of the inhibition circle of 20 wt% Ag-TiO_2_/ZIF-8 is larger than that that of 4 wt% Ag-TiO_2_, ZIF-8, TiO_2_/ZIF-8 (Figure 5), which proves the antibacterial activity was significantly improved by loading Ag-TiO_2_ and there is a synergetic effect between Ag-TiO_2_ and ZIF-8.

#### 3.2.2 Growth curve of bacteria

**FIGURE 6:**
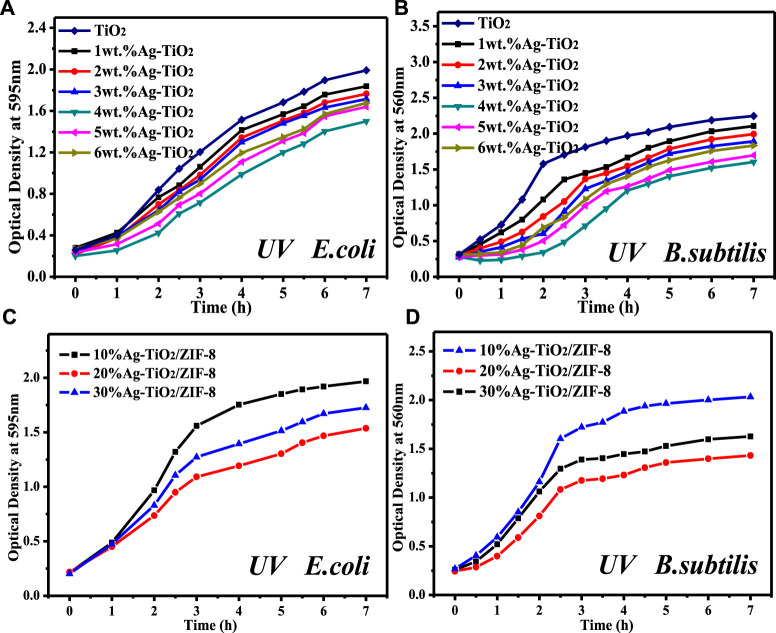
Growth curves of two kinds of bacteria under UV light **(A,C)**: *E. coli*; **(B,D)**: *B.subtilis*.

In order to investigate the effect of the prepared materials on bacterial growth, the growth curves of two types of bacteria were measured. Through preliminary experimental exploration, a loading of 30 mg Ag was selected to prepare 0.2 mg/L Ag-TiO_2_ liquid culture medium. As revealed in Figure 6 and Supplementary Figure S9, pure TiO_2_ inhibited the growth of bacteria, which is consistent with the photocatalytic antibacterial mechanism of TiO_2_ (Jing et al., 2020). A comparative study of Ag-TiO_2_ from 1 wt% to 6 wt% reveals that 4 wt% Ag-TiO_2_ shows the strongest inhibitory effect on bacterial growth, and the smallest OD value was measured at 7 h under dark and UV conditions. In addition, it was found that the high concentration of Ag-TiO_2_/ZIF-8 (0.2 mg/L) could completely inhibit the growth of bacteria used in experiments. Therefore, 15 mg of Ag-TiO_2_/ZIF-8 was chosen to make 0.1 mg/L of liquid medium. Figure 6C, D and Supplementary Figure S10 show that 20 wt% Ag-TiO_2_/ZIF-8 has the best antibacterial activity with the lowest OD values measured at 7 h under dark and UV conditions. When the amount of Ag-TiO_2_/ZIF-8 is half of Ag-TiO_2_, the OD measured at 7 h is smaller, and the concentration of bacteria solution is lower, indicating that the antimicrobial activity of Ag-TiO_2_/ZIF-8 is superior to that of Ag-TiO_2_, and Ag-TiO_2_ has a positive effect on improving the antibacterial activity of Ag-TiO_2_/ZIF-8. These findings are identical to those of the ZOI analysis.

#### 3.2.3 Minimum bactericidal concentration

The MBC of the antibacterial agent refers to the minimum concentration needed to kill 99.9% of the test microorganisms. According to the experiment results of ZOI and bacteria growth curves, 20 wt% Ag-TiO_2_/ZIF-8 has the optimum antibacterial activity. Therefore, 20 wt% Ag-TiO_2_/ZIF-8 was chosen to explore a series of MBCs under dark and UV conditions.

**FIGURE 7:**
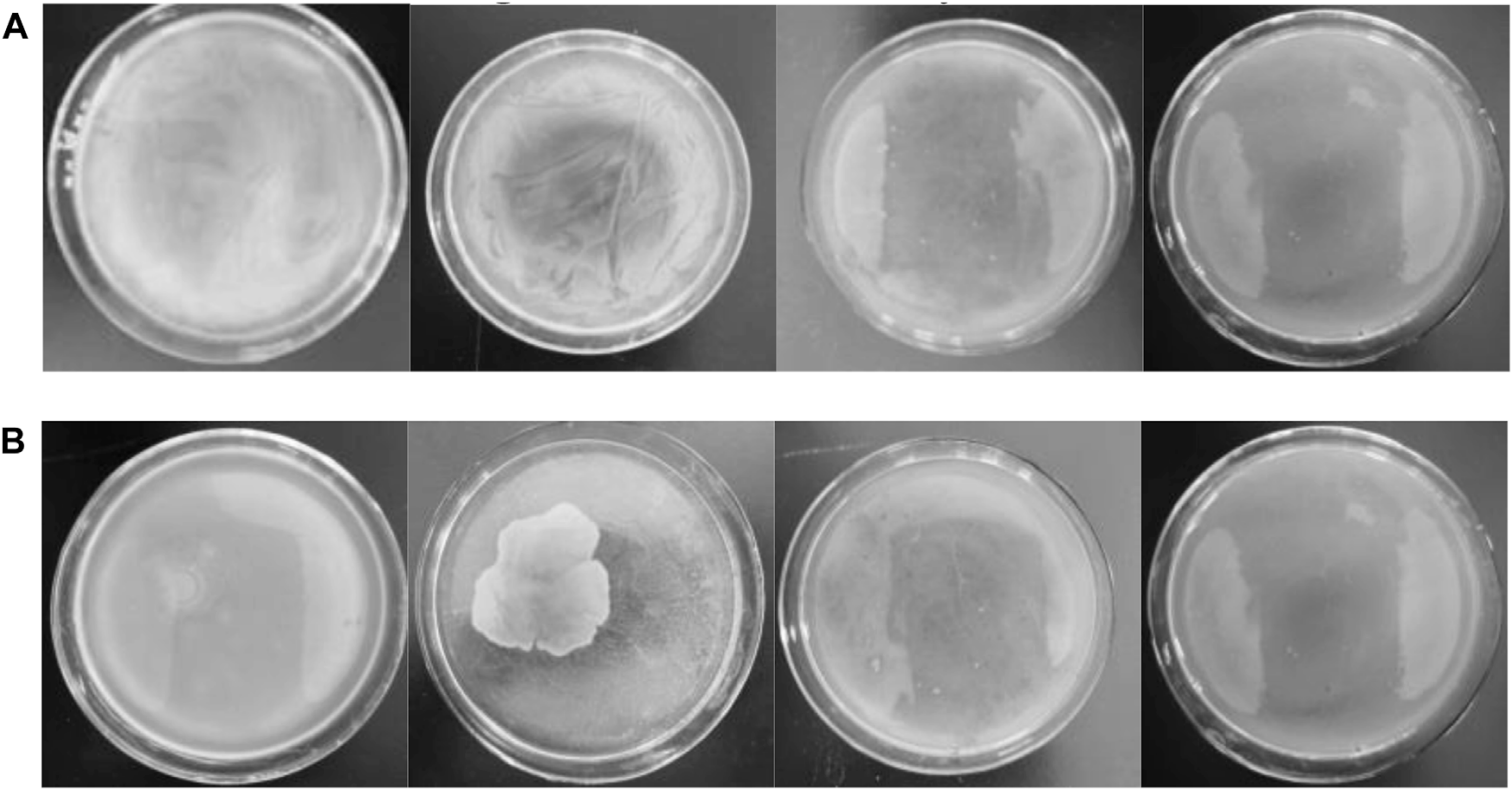
Images of MBC of 20 wt% Ag-TiO_2_/ZIF-8 against *E coli* **(A)** and *B subtilis* **(B)** with the concentration of antibacterial agent increasing from left to right (3.50、3.65、3.70 and 3.80 mg/L) under UV light.

As the concentration of antimicrobial agent increased from left to right, the last LB agar plate is almost free of bacteria after 24 h under dark and UV conditions (Figure 7 and Supplementary Figure S11), demonstrating that Ag-TiO_2_/ZIF-8 possesses excellent antimicrobial activity. The MBS of Ag-TiO_2_/ZIF-8 against *E. coli* and *B. subtilis* are 3.65 and 3.50 mg/L under UV light, which are lower than those under dark conditions (3.80 and 3.70 mg/L), showing the antibacterial activity under UV light is more apparent, and the bactericidal activity against *B. subtilis* is stronger. These results are corroborated by the findings of ZOI and bacterial growth curve analysis.

#### 3.2.4 Antibacterial durability

**FIGURE 8:**
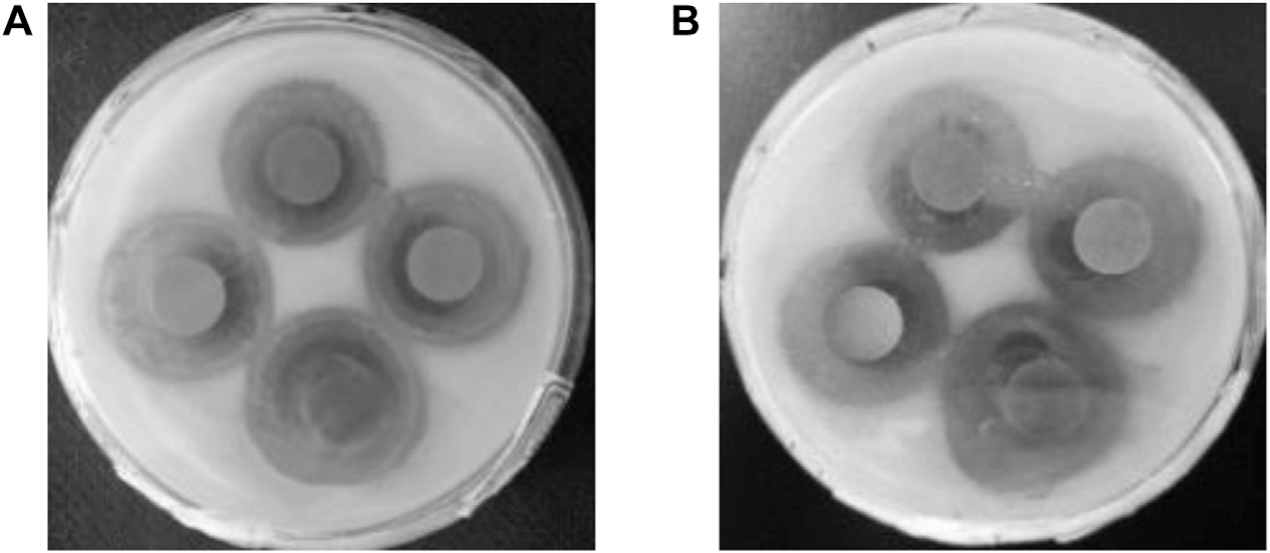
Images of ZOI of Ag-TiO_2_/ZIF-8 against *B subtilis* after 1 day **(A)** and 5 months **(B)** under UV light.

Antibacterial durability is an important indicator of the quality of antimicrobial agents. The ZOI was observed after 5 months of further incubation (Figure 8). The ZOI can visually expand outward while the contours are still noticeable. There are no colonies around, and the antibacterial activity does not diminish after 5 months, indicating the excellent antibacterial durability of Ag-TiO_2_/ZIF-8. Due to its good antimicrobial durability, once it is put into use, it helps to reduce the cost of use. So, Ag-TiO_2_/ZIF-8 is expected to become a long-lasting antimicrobial agent.

### 3.3 Probable antibacterial mechanism

Based on the above experimental results of antibacterial activity evaluation, it can be concluded that the antibacterial activity of Ag-TiO_2_/ZIF-8 is better than that of Ag-TiO_2_ and ZIF-8. The possible antibacterial mechanism of Ag-TiO_2_/ZIF -8 was proposed as follows.

(i) When Ag-TiO_2_/ZIF-8 is added to a solution containing bacteria, it can release Ag^+^ and Zn^2+^ with low toxicity and biological antibacterial activity, these metal ions can come into direct contact with the bacteria and bind to component proteins in cell walls and membranes, denaturing and inactivating the component proteins (Chen et al., 2023; Wang et al., 2023). This causes the outer layer to lose its protection and the bacteria burst and die (Mariappan et al., 2022; Sun et al., 2023). In addition, the interaction between Ag^+^ and DNA structures can prevent bacterial reproduction (Cao et al., 2020; Liu et al., 2022). (ii) TiO_2_ itself has some photocatalytic antibacterial activity. When the energy provided by UV radiation is greater than 3.2 eV, the photogenerated electrons (e_CB_^−^) and holes (h_VB_^+^) generated by TiO_2_ react with water and oxygen to form free ROS, which kill bacteria by destroying cell walls and solidifying viral proteins (Zerjav et al., 2017). Moreover, when TiO_2_ is combined with Ag and ZIF-8 to form a ternary complex, the Ag^+^ and Zn^2+^ in the material can actively inhibit the electron-hole pair recombination and further improve the photocatalytic antibacterial activity (Yang et al., 2019). And Ag-TiO_2_ can also work in dark conditions where bacteria tend to multiply, so the application field of antibacterial agent will be further expanded without the limitation of light sources. (iii) On account of the high BET surface area and porous structure of ZIF-8, more Ag-TiO_2_ is uniformly dispersed on its surface, which increases the contact area between the bactericidal agent and bacteria, thereby improving the antibacterial activity. In addition, Ag^+^ and Zn^2+^ can be slowly released from Ag-TiO_2_/ZIF-8 to achieve a long-term antibacterial effect. In summary, the high antibacterial activity of Ag-TiO_2_/ZIF-8 comes from the synergetic effect between Ag-TiO_2_ and ZIF-8.

## 4 Conclusion

In this paper, Ag-TiO_2_/ZIF-8 ternary with excellent biological antibacterial activity was synthesized by solvothermal method. To evaluate its antibacterial activity against *E. coli* and *B. subtilis*, the experiments such as zone of inhibition, growth curve of bacteria, minimum bactericidal concentration and antibacterial durability were executed. The experimental results demonstrated that 20wt% Ag-TiO_2_/ZIF-8 exhibits the best antibacterial activity under dark and UV conditions, where Ag-TiO_2_ and ZIF-8 had a synergistic effect on antibacterial activity. Since its outstanding antibacterial activity against two ubiquitous bacteria, Ag-TiO_2_/ZIF-8 is a broad-spectrum antibacterial agent. In addition, compared with other antimicrobial materials, the antimicrobial effect of Ag-TiO_2_/ZIF-8 has good stability and durability, and can maintain its antimicrobial activity for more than 5 months without reducing its effectiveness (Liu et al., 2021; Wang et al., 2021). Therefore, Ag-TiO_2_/ZIF-8 is expected to become a promising biological antibacterial material for electrical appliances, furniture coatings, medical facilities and other living fields.

## Data Availability

The raw data supporting the conclusions of this article will be made available by the authors, without undue reservation.
