# Supplementary material for: Synthesis and antibacterial activities of Ag-TiO2/ZIF-8
Source: Front Bioeng Biotechnol. 2023 Jul 27;11:1221458. doi: 10.3389/fbioe.2023.1221458 (PMC10415108; doi:10.3389/fbioe.2023.1221458)
Supplement: Supplementary file 1 [file DataSheet1.docx]

### [Supporting Information](https://www.baidu.com/link?url=fvGSBq5NeKyy7mgnEN4Mdo5K25XY-pAjB3xJo2JtqpUKMt-XZuwP-yUlzOsSBarA9Ze0Xv-lrQagUc_Wxh6k2_&wd=&eqid=f72f6e030015a517000000025e5c6938)

###

Fig. S1. FT-IR spectra of 20 wt.%Ag-TiO_2、_4 wt.% Ag-TiO_2_


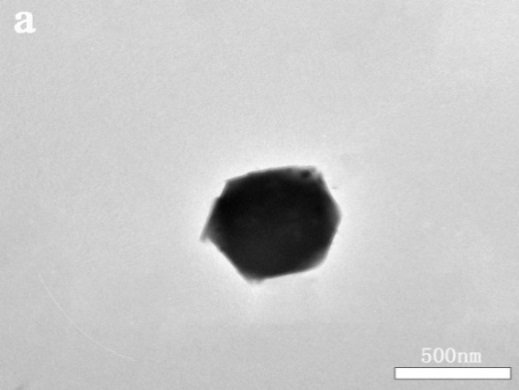

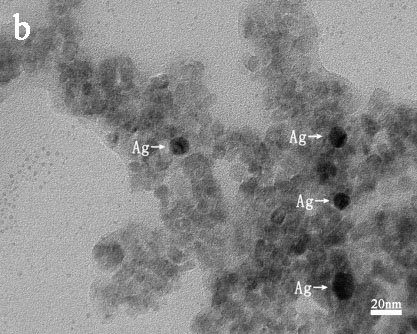

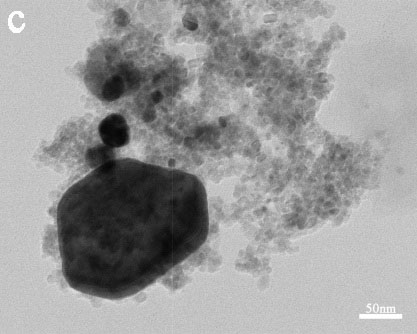

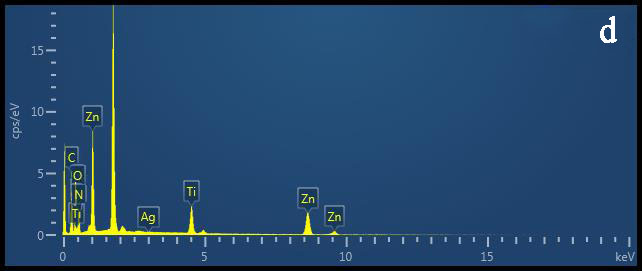

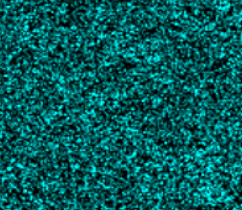

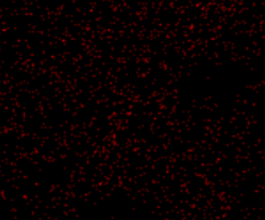

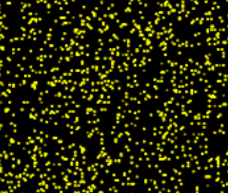


C

O

g

f

e

Zn

j


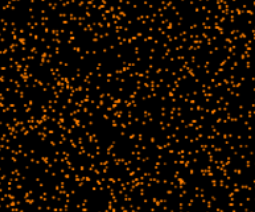

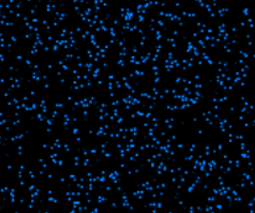

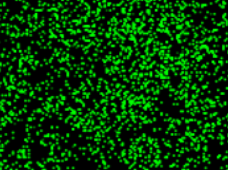


j

N

Ag

i

h

Ti

Fig. S2 TEM images of ZIF-8 (a), 4 wt.% Ag-TiO_2_ (b), 20 wt.%Ag-TiO_2_/ZIF-8 (c) and EDS spectrum of 20 wt.% Ag-TiO_2_/ZIF-8 (d)，EDS images of 20 wt.%Ag-TiO_2_/ZIF-8(e-j)


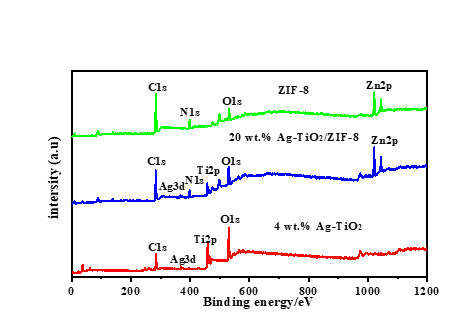


Fig. S3 XPS survey spectrum of TiO_2_, ZIF-8, 4 wt.%Ag-TiO_2_ and 20 wt.% Ag-TiO_2_/ZIF-8

Fig. S4 Nitrogen adsorption-desorption isotherms (a) and pore size distribution

profiles (b) of 4 wt.%Ag-TiO_2,_ ZIF-8 and the different loadings of Ag-TiO_2_/ZIF-8

(A)
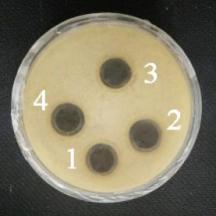

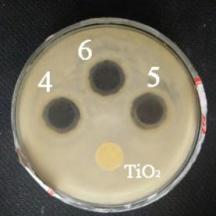
 (B)
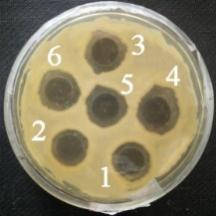


Fig. S5 Images of ZOI of pure TiO_2_ and 1 wt.%～6 wt.% Ag-TiO_2_

against E.coli (A) and B.subtilis (B) at dark condition

(A)
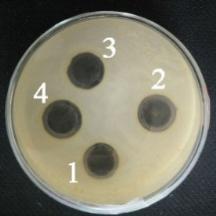

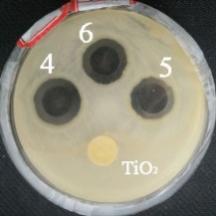
 (B)
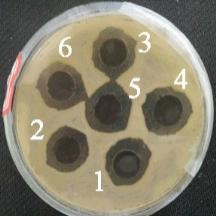


Fig. S6 Images of ZOI of pure TiO_2_ and 1 wt.%～6 wt.% Ag-TiO_2_

against E.coli (A) and B.subtilis (B) under UV light

(A)
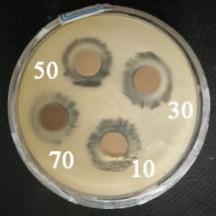

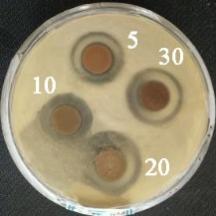
 (B)
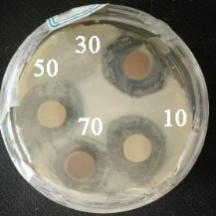

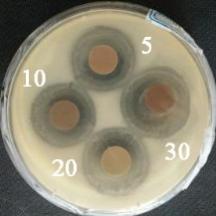


Fig. S7 Images of ZOI of 5wt.%~70wt.% Ag-TiO_2_/ZIF-8 against E.coli (A)

and B.subtilis (B) at dark condition

(C)
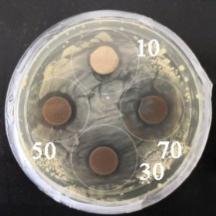

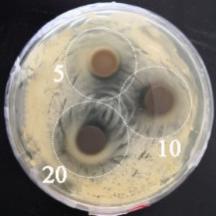
 (D)
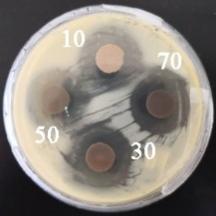

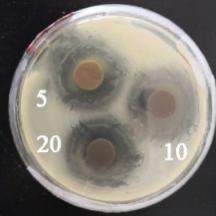


Fig. S8 Images of ZOI of 5wt.%~70wt.% Ag-TiO_2_/ZIF-8 against E.coli (C)

and B.subtilis (D) under UV light.

Fig. S9 Growth curves of two kinds of bacteria at dark condition, Ecoli (a) and B.subtilis (b).

Fig. S10 Growth curves of two kinds of bacteria at dark condition, Ecoli (a) and B.subtilis (b).

(A)
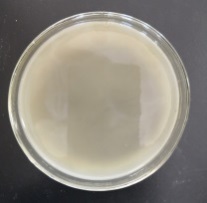

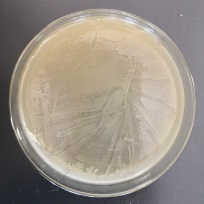

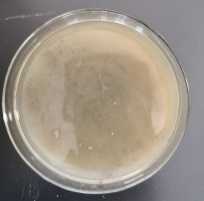

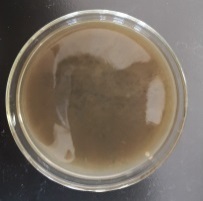


(B)
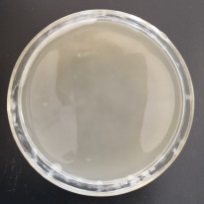

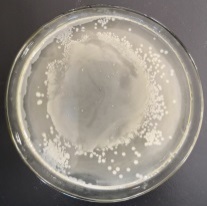

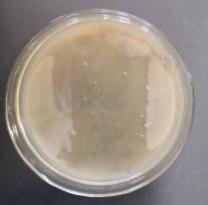

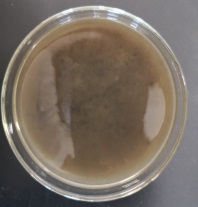


Fig. S11 Images of MBC of 20 wt.% Ag-TiO_2_/ZIF-8 against E.coli (A)

and B.subtilis (B) with the concentration of antibacterial agent increasing from left to right (3.50、3.65、3.70 and 3.80mg/L) at dark condition

### Tab. S1 Ag-TiO_2_/ZIF-8 Ternary Composites with Different Ag-TiO_2_ Modifications (Abbreviated as "F")

| **Material** | **5 wt.%F** | **10 wt.%F** | **20 wt.%F** | **30 wt.%F** | **50 wt.%F** | **70 wt.%F** |
| --- | --- | --- | --- | --- | --- | --- |
| Zn(NO_3_)_2_.6(H_2_O) (mg) | 477.8 | 477.8 | 477.8 | 477.8 | 477.8 | 477.8 |
| 4 wt.% Ag-TiO_2_ (mg) | 8.8 | 18.7 | 42.0 | 72.0 | 168.0 | 392.0 |

### Tab. S2 Specific surface area, average pore size and pore volume of different materials

| **Samples** | **S_BET_ (m^2^**▪**g^–1^)** | **V_p_ (cm^3^**▪**g^–1^)** | **D_p_ (nm)** |
| --- | --- | --- | --- |
| ZIF-8 | 637.9 | 0.61 | 3.84 |
| 4 wt.% Ag-TiO_2_ | 173.2 | 0.26 | 6.10 |
| 20 wt.% Ag-TiO_2_/ZIF-8 | 474.6 | 0.53 | 4.46 |

Tab. S3 Diameters of ZOI of 1wt.%~6wt.% Ag-TiO_2_ (“A-T”) (mm)

| **Samples** | **1wt.%A-T** | | **2wt.%A-T** | **3wt.%A-T** | **4wt.%A-T** | **5wt.%A-T** | **6wt.%A-T** |
| --- | --- | --- | --- | --- | --- | --- | --- |
| E.coli (Dark) | | 15.4 | 16.1 | 15.9 | 16.7 | 16.5 | 16.3 |
| B.subtilis ( Dark ) | | 17.8 | 18.2 | 18.7 | 20.4 | 19.6 | 19.0 |
| E.coli  (UV) | | 16.1 | 16.8 | 17.0 | 17.6 | 17.4 | 17.2 |
| B.subtilis (UV) | | 20.2 | 20.6 | 21.0 | 22.8 | 22.2 | 21.6 |

Tab. S4 Diameters of ZOI of 5wt.%~70wt.% Ag-TiO_2_/ZIF-8 (“F”) (mm)

| **Samples** | **5 wt.%F** | **10 wt.% F** | **20 wt.% F** | **30 wt.%F** | **50 wt.%F** | | **70 wt.%F** | |
| --- | --- | --- | --- | --- | --- | --- | --- | --- |
| E.coli (Dark) | 24.2 | 25.1 | 25.9 | 25.0 | | 24.0 | | 23.4 |
| B.subtilis ( Dark ) | 29.4 | 30.6 | 31.8 | 30.0 | | 29.4 | | 28.8 |
| E.coli  (UV) | 25.8 | 26.0 | 26.2 | 25.3 | | 24.9 | | 23.8 |
| B.subtilis (UV) | 32.0 | 32.4 | 33.0 | 31.8 | | 31.4 | | 30.0 |
